# Japan's lack of accountability for conducting research on deadly pathogens

**DOI:** 10.1002/jgf2.292

**Published:** 2020-01-05

**Authors:** Yasuharu Tokuda

**Affiliations:** ^1^ Muribushi Okinawa for Teaching Hospitals Okinawa Japan

During the Second World War, scientists and physicians of Unit 731 of the Japanese Imperial Army developed bioweapons, including plague, tularemia, and anthrax. Human experiments were conducted by these bioweapons in Harbin City, which had been occupied by Japan in the Chinese continent. The antihumanitarian experiment killed thousands of people as subjects.

After the war, however, these scientists were not formally accused at the Tokyo war trial because they forwarded data of the experiments to the US military group for avoiding criminal charges against them. The scientists claimed that they conducted research to counter possible attack of bioweapons developed by other countries.^1^

In addition to universities and academic societies that were involved with the Unit 731 activity, the Japanese government still has not formally recognized this unethical activity. No ethics education based on reflecting the conduct of Unit 731 was provided to medical students and residents in Japan. It is still true after I pointed this issue 25 years ago,^2^ when scientists and physicians of Japanese Aum Shinrikyo Cult developed chemical and biological weapons and used sarin gas and killed many people in Tokyo subway. The government and the involved universities of Japan lack accountability.

According to news by Nature journal,^3^ the Japanese government recently imported Ebola and other deadly viruses. The government claimed that they would use them as preparation against possible outbreak of the infectious diseases during the 2020 Tokyo Olympic. Local people in Nagasaki City make protests against the collection of these viruses in an institution of Nagasaki University. The Japanese government should show accountability and formally apologize victims’ family for the truth of the Unit 731 activity before conducting any future research on deadly pathogens. Unless this would be done, the Japanese government has no ethical qualifications to do it.

## CONFLICT OF INTEREST

The author has stated explicitly that there are no conflicts of interest in connection with this article.
